# Insufficient or excessive exercise activities are associated with suboptimal treatment outcomes in patients with psoriasis: a longitudinal study in shanghai, China

**DOI:** 10.1080/07853890.2026.2687187

**Published:** 2026-06-15

**Authors:** Quanruo Xu, Ruiqi Cai, Jinrong Lu, Yuning Ding, Zhen Duan, Xiuqi Zhang, Xiangjin Gao, Rui Zhang, Ruiping Wang

**Affiliations:** ^a^ Clinical Research Center, Shanghai Skin Diseases Hospital, School of Medicine, Tongji University, Shanghai, China; ^b^ School of Public Health, Shanghai University of Traditional Chinese Medicine, Shanghai, China; ^c^ School of Medicine, Tongji University, Shanghai, China

**Keywords:** Psoriasis, exercise activity, MET, treatment outcome, PASI_50_

## Abstract

**Background:**

Physical activity’s impact on psoriasis treatment outcomes is unclear. This study examines associations between exercise levels and treatment response in psoriasis patients.

**Methods:**

In a longitudinal study of 1,116 psoriasis patients (2022–2024), physical activity was assessed *via* MET-min/week and categorized as inactive (<150), low (150–<500), moderate (500–<1000), or high (≥1000). Severity (PASI/BSA) and PASI_50_ response were evaluated at baseline, week 4, and week 8. Analyses used logistic regression and restricted cubic splines.

**Results:**

Among participants (74.4% male, mean age 46.1), week 8 PASI_50_ rates were 61.2% (inactive), 75.1% (low), 69.9% (moderate), and 66.7% (high). Low activity was linked to higher odds of PASI_50_ (adjusted OR = 1.58; 95% CI: 1.01–2.79) versus inactivity. A J-shaped nonlinear association emerged (*p* = 0.023): response probability rose rapidly at low exercise levels, peaked at 150–500 MET-min/week, the benefit plateaued or attenuated at higher intensities (≥1000 MET-min/week).

**Conclusion:**

Moderate physical activity (150–500 MET-min/week) is associated with better psoriasis treatment outcomes, with diminishing benefits at very low or high levels. Findings support personalized, intensity-based exercise recommendations.

## Introduction

Psoriasis is a chronic, immune-mediated inflammatory skin disorder, characterized by erythematous, scaly plaques, which affects approximately 2-3% of the global population [1,2]. Beyond its cutaneous manifestations, psoriasis is associated with substantial psychosocial impairment and systemic comorbidities, including psoriatic arthritis, cardiovascular diseases, and metabolic syndrome, collectively contributing to significant healthcare burdens and diminished quality of life [3]. Current therapeutic strategies encompass topical agents, phototherapy, conventional systemic drugs, small molecules and biologic agents. While these treatments can be effective, not all patients achieve satisfactory or sustained remission. Issues such as high treatment costs, potential side effects, and variable individual responses highlight the urgent need for identifying safe, accessible, and effective adjunctive approaches to enhance clinical outcomes [4,5].

Emerging evidence suggests that lifestyle interventions, particularly physical activity, may serve as a promising adjunct in psoriasis management. Regular exercise is well established to confer broad anti-inflammatory and immunomodulatory benefits, including reduced circulating levels of pro-inflammatory cytokines such as TNF-α and IL-17, elevated anti-inflammatory mediators such as IL-10, and improved metabolic parameters-all of which are relevant to the pathogenesis of psoriasis [6,7]. Large-scale epidemiological studies have demonstrated an inverse association between self-reported physical activity and the incidence or severity of psoriasis [8–10]. For instance, a prospective cohort study by Frankel et al. found that physically active women had a significantly lower risk of developing psoriasis [8]. Several observational studies have further explored this relationship from both preventive and therapeutic perspectives. In a cross-sectional survey, Balato et al. observed that psoriasis was significantly less common among individuals engaged in regular sports activities compared with sedentary controls, and that physically active psoriatic patients more often reported a positive influence of exercise on their disease course [11]. Similarly, a study of professional soccer players by Megna et al. [12] showed that regular, intense physical activity was associated with better disease control, suggesting that consistent exercise may confer benefits even in individuals with high physiological demands. A comprehensive review by Wilson et al. [13] summarized the growing body of evidence linking physical activity to psoriasis outcomes, highlighting shared pathophysiological pathways such as inflammation, oxidative stress, and epigenetic modifications, while also noting methodological limitations in existing studies. In addition, systematic reviews have indicated that exercise interventions may improve disease severity and quality of life in patients with psoriasis [14,15]. Collectively, these studies support the potential role of physical activity in psoriasis management, while underscoring the need for more rigorous, dose–response investigations.

However, the relationship between physical activity and psoriasis may not be universally beneficial. Emerging evidence suggests a potential J-shaped or U-shaped association, wherein excessively high-activity exercise could act as a physiological stressor, potentially attenuating the anti-inflammatory benefits and even exacerbating disease activity in some individuals. A large cohort study found that while moderate physical activity was associated with a decreased risk of incident psoriasis, individuals engaging in the highest levels of vigorous exercise (≥5 h per week) exhibited a non-significant increased risk, hinting at a potential threshold effect beyond which benefits may plateau or reverse [8]. Furthermore, a mechanistic study by Gjevestad et al. demonstrated that an acute bout of high-activity exercise could transiently increase circulating levels of pro-inflammatory cytokines, including IL-6 and IL-8, in untrained individuals [16]. These studies underscore the complexity of the relationship and highlight the need to precisely define the optimal dose of exercise, as both insufficient and excessive activity might yield suboptimal outcome [17].

Despite these encouraging observations, critical evidence gaps persist in the field of psoriasis and physical activity research. First, most previous studies classified physical activity as a binary variable (active/inactive), which cannot reflect the continuous and dose-dependent characteristics of exercise activity, leading to an inability to accurately identify the optimal exercise intensity for psoriasis patients [17,18]. Second, few studies have used metabolic equivalent task minutes (MET-min/week) to quantify exercise activity, resulting in poor comparability of research results across studies. Third, the non-linear dose-response relationship between exercise activity and psoriasis treatment efficacy has not been fully elucidated, and traditional linear regression analysis cannot capture the potential non-linear patterns (e.g. J-shape or U-shape) of this association.

Based on the aforementioned gaps, this longitudinal observational study aims to: (1) Quantitatively assess exercise activity in psoriasis patients using MET-min/week, and classify exercise intensity into four graded categories instead of binary classification, to accurately reflect the dose characteristics of exercise; (2) Explore the non-linear association between exercise activity and PASI_50_ treatment response using restricted cubic spline (RCS) analysis.

## Methods

### Study population

In this study, patients with psoriasis were enrolled from the Shanghai Skin Disease Hospital between 2022 and 2024. All participants provided written informed consent. Eligible patients were aged ≥ 18 years with a clinical diagnosis of psoriasis vulgaris in accordance with global diagnostic and treatment guidelines [19]. All patients were newly initiated on their respective treatments at baseline (week 0); patients who had received the same treatment for > 4 weeks before enrollment were excluded. Exclusion criteria included planned relocation within one year, inability to provide informed consent, and the presence of significant neurological or psychiatric disorders. The study protocol was approved by the Institutional Ethical Review Board of Shanghai Skin Diseases Hospital (2022-25). We have registered this study in the Chinese clinical trial registry (ChiCTR2200066894), and performed it in line with the Declaration of Helsinki and STROBE guidelines.

### Sample size

Data was extracted from the psoriasis cohort established at Shanghai Skin Diseases Hospital during 2022 and 2024, the detailed information regarding the psoriasis cohort was described in our previous publication [20]. A total of 1160 patients with psoriasis were initially recruited from January 2022 to December 2024. During the 8-week follow-up, 44 patients were lost (3.8%), including 21 patients who moved out of Shanghai, 15 patients who withdrew their informed consent, and 8 patients with unknown reasons. After excluding these patients, the final analytical sample comprised 1,116 participants (96.2%). Missing data for baseline covariates were minimal (<2% for any variable).

### Data collection

Data were collected through a questionnaire administered by dermatologists during patients’ hospital visits. The questionnaire encompassed the following domains: (1) Demographic features: gender, age, educational status, monthly income, and body mass index (BMI); (2) Lifestyle habits: tobacco smoking and alcohol consumption; (3) Physical activity assessment: the physical activity status of patients was investigated using a validated Chinese version of the International Physical Activity Questionnaire (IPAQ)-Short Form (Cronbach’s α = 0.82, test-retest reliability = 0.78) [21,22]. The questionnaire included four categories of physical activity: moderate-intensity exercise (e.g. brisk walking, swimming), high-intensity exercise (e.g. basketball, football), transport-related physical activity (e.g. walking or cycling for commuting), and household physical activity (e.g. cleaning, cooking). Patients were asked to report monthly frequency and average duration per session of each activity, and the investigators verified the reported information with the patients during the face-to-face investigation to reduce reporting errors; (4) Psoriasis severity and treatment evaluation: Body Surface Area (BSA), Psoriasis Area and Severity Index (PASI) were evaluated at baseline, week 4, and week 8.

### Definition and classification

Physical activity levels were quantified using the metabolic equivalent of task (MET), defined as the ratio of working to resting metabolic rate, with one MET equivalent to 3.5 mL O_2_/kg/min. Weekly energy expenditure (MET-min/week) was calculated using the formula: MET-min/week = MET value × frequency (sessions/month) × duration (minutes/session) ÷ 4 based on patient-reported data across the above four domains: moderate-activity exercise (METs = 4.0), high activity exercise (METs = 8), transport-related activities (METs = 4), and household activities (METs = 3). MET values for each activity were derived from the Compendium of Physical Activities, a standardized and widely used reference [21].

Patients were then classified into four exercise activity groups based on established thresholds: inactive (MET ≤ 150), low activity (150 ≤ MET < 500), moderate activity (500 ≤ MET < 1000), and high activity (MET ≥ 1000). The threshold of 150 MET-min/week aligns with the minimum physical activity level recommended by the World Health Organization for maintaining general health [22]. The 500 MET-min/week cutoff represents a recognized moderate level of activity associated with substantial health gains in epidemiological studies [23]. The 1000 MET-min/week threshold is commonly used to define a high level of energy expenditure in research on physical activity and chronic diseases [24].

Psoriasis area and severity index (PASI) was employed to assess skin lesion severity, with a higher value represents a more severe condition (0[better]-72[worse]). The PASI_50_ were defined as patients achieving > 50% PASI score improvement after treatment, which was calculated as [(PASI at baseline - PASI at week t)/PASI at baseline] × 100%. Body surface area (BSA) represent the affected body surface, with a higher value represents a more severe condition (0%[better] − 100%[worse].)

In this study, we defined a smoker as an individual who consumed ≥100 cigarettes in their lifetime and a drinker as someone who consumed alcohol >2 times per week for at least 6 months [25]. Age was categorized into <35, 35–45, 46–60, and >60 years. Education level was classified as primary school or lower (0–6 years), junior high (7–9 years), senior high (10–12 years), and college and above (>12 years) based on their completed years of schooling. Monthly income was divided into <3000, 3000–5000, 5001–10,000 and >10,000 RMB. Body mass index (BMI) was calculated as weight (kg) divided by squared height (m^2^) and then categorized as <23.9 (low or normal weight), 24.0–28.0 (overweight), and >28.0 (obesity).

### Statistical analysis

Data analysis was performed using R software (version 4.2.2). For the low missing rate (<5%) of physical activity assessment, we used the multiple imputation (MI) method and generated 20 imputed datasets using the MICE package in R software to handle missing data. In this study, continuous variables with normal distribution were presented as mean and standard deviation (SD) and compared using Student’s t-test. Skewed distributed continuous variables were summarized as median (interquartile range, IQR) and compared using the Mann-Whitney U test. Categorical variables were described as frequencies and percentages (%), with group differences assessed by the chi-square test.

The association between exercise activity and PASI_50_ achievement was evaluated using multivariable logistic regression, with results expressed as odds ratios (OR) and 95% confidence intervals (CI). The assumptions of the logistic regression model were tested before analysis: (1) There was no serious multicollinearity among independent variables (variance inflation factor, VIF < 3); (2) The linear relationship between the logit of the dependent variable and continuous independent variables (e.g. age, BMI, baseline PASI score) was verified using the Box-Tidwell test; (3) The model fit was evaluated using the Hosmer-Lemeshow test (*p* = 0.68 for week 8, *p* = 0.72 for week 4), indicating a good fit between the logistic regression model and the observed data.

To examine the potential nonlinear relationship between continuous MET-min/week and the probability of achieving PASI_50_, the restricted cubic spline (RCS) models were fitted using the rms package in R software with the adjustment for gender, smoking, alcohol drinking, baseline PASI score, treatment and GAD-7. The uncertainty of the RCS model was reflected by 95% confidence bands, and the statistical significance of the nonlinear association was tested using the likelihood ratio test (LRT) by comparing the RCS model with the linear regression model. Subgroup analysis was performed to show the distribution difference of MET values by gender and age. All statistical tests were two-tailed, and a p-value < 0.05 was considered statistically significant.

## Results

In this study, 1116 psoriasis patients were enrolled, including 830 (74.4%) males and 286 (25.6%) females, with a mean age of 46.1 years (SD =16.3). Approximately 44% of participants had college or higher education. Monthly income distribution showed that 45.7% earned 5,001–10,000 RMB. 74.1% patients were married. The mean BMI was 25.0 kg/m^2^ (SD =4.0), with 452 patients (40.9%) classified as low/normal weight, 437 (39.1%) as overweight, and 217 (19.7%) as obesity. The prevalence of tobacco smoking and alcohol drinking was 47.3% and 17.8%, respectively. Baseline disease severity median scores were 11.2 (IQR: 7.9–16.8) for PASI and 15.0 (IQR: 9.5–27.0) for BSA, respectively (Table 1).

**Table 1.** Characteristics of patients in different exercise activity intensity^a^.

Characteristics	Total (*n* = 1116)	Baseline features of patients in different groups			
		MET < 150 (*n* = 67)	150 ≤ MET < 500 (*n* = 539)	500 ≤ MET < 1000 (*n* = 408)	MET ≥ 1000 (*n* = 102)
Gender (n,%)^‡^					
Male	830 (74.4)	53 (6.4)	412 (49.6)	282 (34.0)	83 (10.0)
Female	286 (25.6)	14 (4.9)	127 (44.6)	126 (43.9)	19 (6.7)
Age group, (n, %)					
<35 years	286 (25.6)	16 (5.6)	129 (45.1)	116 (40.6)	25 (8.7)
35–45 years	250 (22.4)	15 (6.0)	137 (54.8)	74 (29.6)	24 (9.6)
46–60 years	280 (25.1)	13 (4.6)	139 (49.6)	104 (37.1)	24 (8.6)
>60 years	299 (26.9)	23 (7.7)	134 (44.8)	113 (37.8)	29 (9.7)
education, (n, %)					
Primary and lower	144 (12.9)	7 (4.9)	58 (40.3)	65 (45.1)	14 (9.7)
Junior high	231 (20.7)	15 (6.5)	116 (50.2)	80 (34.6)	20 (8.7)
Senior high	249 (22.3)	17 (6.8)	108 (43.4)	99 (39.8)	25 (10.0)
College and above	491 (44.0)	28 (5.7)	257 (52.3)	165 (33.2)	41 (8.8)
Monthly income (RMB), (n, %)					
<3000	137 (12.3)	10 (7.3)	62 (45.3)	55 (40.1)	10 (7.3)
3000–5000	307 (27.5)	14 (4.6)	146 (47.6)	114 (37.1)	33 (10.7)
5001–10000	510 (45.7)	33 (6.5)	248 (48.7)	182 (35.6)	47 (9.2)
>10000	162 (14.5)	10 (6.2)	83 (51.2)	57 (35.2)	12 (7.4)
Residency status, (n,%)					
Urban	704 (63.1)	43 (6.1)	345 (49.1)	251 (35.6)	65 (9.2)
Rural	412 (36.9)	24 (5.8)	194 (47.1)	157 (38.1)	37 (9.0)
Marital status, (n, %)^‡^					
Married	826 (74.1)	44 (5.3)	413 (50.0)	290 (35.1)	79 (9.6)
Unmarried	182 (16.3)	10 (5.5)	88 (48.4)	72 (39.6)	12 (6.6)
Divorced/others	107 (9.6)	13 (12.1)	38 (35.5)	45 (42.1)	11 (10.3)
BMI (kg/m^2^), mean SD	25.0 (4.0)	26.5 (5.0)	25.3 (3.9)	24.6 (4.0)	24.3 (3.8)
BMI (kg/m^2^), n (%)^b‡^					
<23.9 (low or normal weight)	452 (40.9)	22 (4.9)	198 (43.8)	185 (40.9)	47 (10.4)
24.0–28.0 (overweight)	437 (39.1)	26 (5.9)	221 (50.6)	149 (34.1)	41 (9.4)
>28.0 (obesity)	217 (19.7)	19 (8.8)	115 (53.0)	70 (17.3)	13 (12.9)
Tobacco smoking, n (%)	528 (47.3)	32 (6.1)	242 (45.8)	199 (37.7)	55 (10.4)
Alcohol drinking, n (%)^‡^	199 (17.8)	22 (11.1)	99 (49.7)	64 (32.3)	14 (6.0)
PASI at week0, median (IQR)^c^	11.2 (7.9–16.8)	11.1 (8.4–15.6)	11.2 (8.0–16.8)	11.3 (7.7–16.9)	11.2 (7.8–16.2)
BSA at week0, median (IQR)^d^	15.0 (9.5–27.0)	14.5 (8.0–24.0)	14.0 (9.5–24.0)	15.0 (10.0–29.0)	17.5 (8.5–31.3)
Psoriasis duration, median (IQR)	12.0(4.0–21.0)	11.0(3.0–22.0)	12.0(4.0–21.0)	12.0(5.0–20.0)	10.0(5.0–19.0)
Treatment, (n, %)^‡^					
Acitretin	121(10.9)	10(8.3)	55(45.5)	41(33.9)	55(12.4)
MTX	283(25.5)	17(6.0)	121(42.8)	119(42.0)	26(9.2)
NB-UVB	234(21.1)	19(8.1)	101(43.2)	94(40.2)	20(8.5)
Benvitimod	29(2.6)	1(3.4)	19(65.5)	5(17.2)	4(13.8)
Biologics	444(40.0)	20(4.5)	241(54.3)	147(33.1)	36(8.1)

MET: metabolic equivalent; SD: standard deviation; RMB: Chinese Yuan; BMI: body mass index; IQR: Interquartile range; PASI: psoriasis area and severity index; BSA: body surface area; PGA: Physician’s Global Assessment.

^a^ Data represented as mean (SD), median (IQR) or frequency (%) as appropriate.

^b^ BMI was calculated as weight in kilograms divided by height in meters squared.

^c^ PASI indicate the severity of psoriasis, with a higher value represents a more severe condition (0[better]-72[worse]).

^d^ BSA indicate the affected body surface, with a higher value represents a more severe condition (0%[better]-100%[worse]).

^‡^ The difference between the patients in different exercise intensity groups was statistically significant.

### Exercise activity distribution and population characteristics

The total 1,116 patients with psoriasis were stratified into 4 exercise activity groups according to MET-minute expenditure, including inactive (MET < 150; *n* = 67 [6.0%]), low (150 ≤ MET < 500; *n* = 539 [48.3%]), moderate (500 ≤ MET < 1000; *n* = 408 [36.6%]), and high (MET ≥ 1000; *n* = 102 [9.1%]). Gender distribution varied significantly across different exercise activity groups (*p* < 0.05), with the highest proportion of female participants observed in the moderate-activity group (126/408 [44.1%]). An inverse relationship was observed between exercise activity and body mass index (BMI), with the higher exercise activity group associated with the lower proportion of BMI > 24.0. Additionally, the prevalence of alcohol consumption was significantly higher in the inactive-activity group (22/67, 32.8%) compared to other groups (99/539, 18.4%; 64/408, 15.7%; 14/102, 13.7%) (*p* < 0.05) (Table 1).

**Figure 1.**
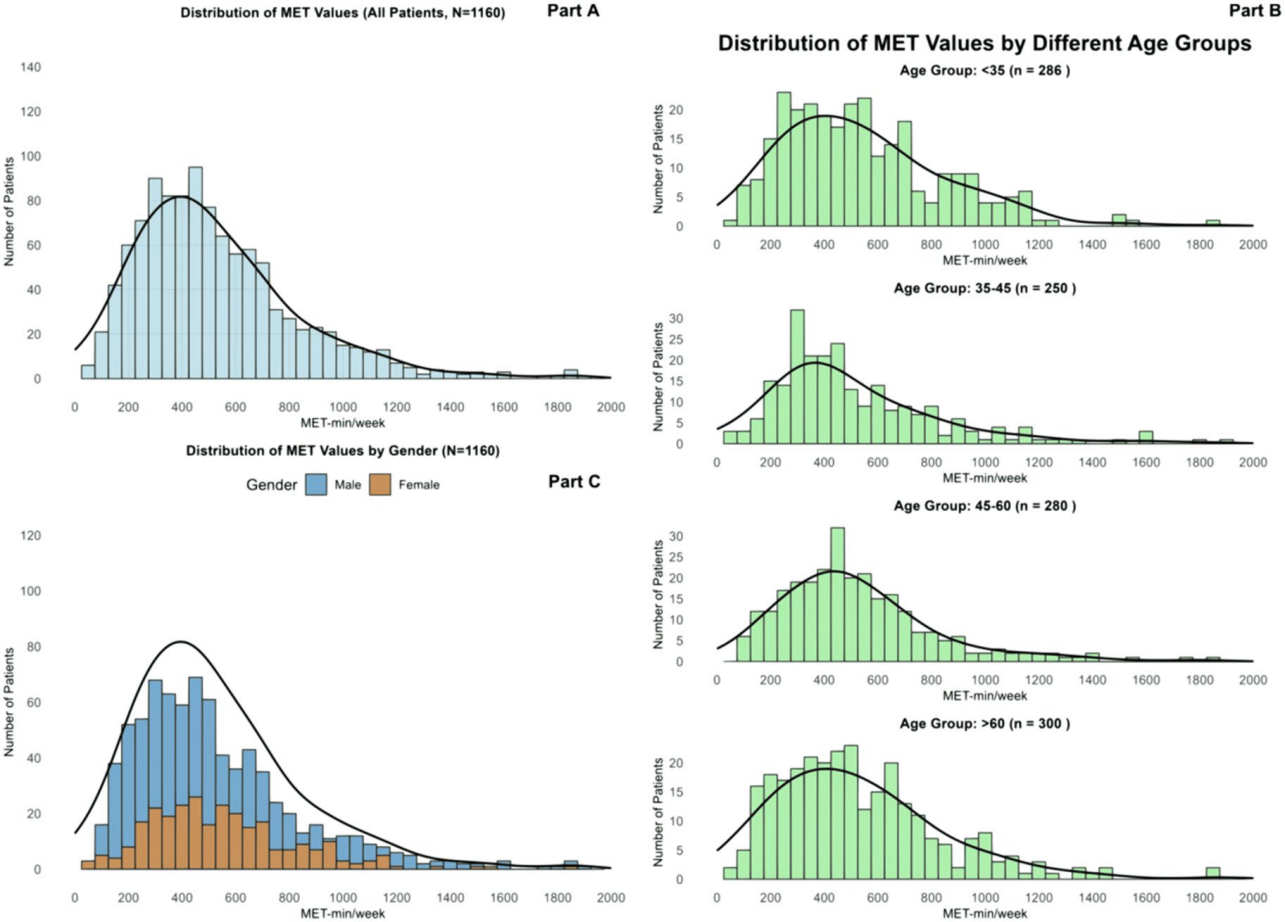
Distribution of MET values among psoriasis patients by overall population, age groups, and gender(Part A. Overall distribution of MET- min/week in all 1,116 patients; Part B. Distribution of MET-min/week in age groups: <35 years (*n* = 286), 35–45 years (*n* = 250), 45–60 years (*n* = 280), and >60 years (*n* = 300); Part C. Distribution of MET-min/week by gender (male vs. female). MET: metabolic equivalent task.

Figure 1 indicated the overall MET-min/week values exhibiting a right-skewed distribution, with the modal value located within the 150 ≤ MET < 500 range. Gender-based analysis revealed significantly higher median MET values among male participants compared to females (580 vs 420 MET-min/week; *p* < 0.01). Patients <35 years (*n* = 286) showed a right-skewed distribution with the highest median value of exercise activity (550 MET-min/week), and the median value of exercise activity decreased in the 35–45 years group (400 MET-min/week) but then increased gradually with the elevation of age, with a median value of 460 MET-min/week for the 46–60 years group and 485 MET-min/week for the >60 years group (Figure 1).

### Treatment outcomes according to exercise activity

At week 4, the median PASI scores across the four exercise groups ranged from 5.6 (IQR: 3.2–10.2) to 6.7 (IQR: 3.9–10.3), with no statistically significant difference observed (*p* > 0.05). The median value of percentage improvement in PASI score ranged from 38.0% to 45.0%, and the PASI_50_ response rate varied from 36.3% to 44.0%, both without significant variations across the four exercise groups. Similarly, no statistically significant differences among the four exercise activity groups were observed in BSA scores, absolute changes in BSA, or percentage improvement in BSA at week 4 (Table 2).

**Table 2.** Therapeutic effects of patients with psoriasis at the 4th and 8th weeks under different exercise intensities.

Characteristics	Treatment effect at week 4				Treatment effect at week 8			
	MET < 150 (*n* = 67)	150 ≤ MET < 500 (*n* = 539)	500 ≤ MET < 1000 (*n* = 408)	MET ≥ 1000 (*n* = 102)	MET < 150 (*n* = 67)	150 ≤ MET < 500 (*n* = 539)	500 ≤ MET < 1000 (*n* = 408)	MET ≥ 1000 (*n* = 102)
Primary outcome								
PASI score, median (IQR)	6.7 (3.9–10.3)	6.0 (3.1–10.5)	5.6 (3.2–10.2)	6.0 (3.3–9.8)	4.1 (1.5–7.2)	3.2 (1.1–6.5)	3.3 (1.6–6.8)	4.0 (1.8–7.9)
Change of PASI score, median (IQR)	4.1 (1.0–8.0)	4.9 (2.0–8.8)	4.3 (1.6–8.0)	3.8 (1.7–8.0)	6.6 (2.6–12.1)	7.3 (4.6–11.8)	6.9 (3.8–11.3)	5.9 (3.5–10.9)
Percentage of PASI change (%), median (IQR)^‡^	38.0 (13.8–67.8)	45.0 (22.6–67.3)	43.5 (17.1–66.7)	39.3 (18.0–66.1)	65.0 (27.9–83.3)	70.0 (50.0–89.1)	66.8 (46.4–85.7)	61.2 (41.5–79.8)
Percentage of PASI_50_, n (%)^‡^	28 (41.8)	237 (44.0)	177 (43.4)	37 (36.3)	41 (61.2)	405 (75.1)	285 (69.9)	68 (66.7)
Secondary outcome								
BSA score, median (IQR)	10.0 (5.0–18.1)	9.0 (4.9–17.0)	10.4 (5.2–18.8)	11.0 (4.9–20.0)	6.0 (1.3–10.5)	5.0 (2.0–11.0)	5.3 (2.0–14.0)	7.0 (2.6–13.1)
Change of BSA score, median (IQR)	2.5 (0.2–8.0)	4.0 (1.0–10.0)	3.5 (0.0–10.0)	3.0 (0.4–8.6)	6.0 (1.5–15.4)	8.0 (4.0–15.1)	7.5 (3.0–15.0)	7.0 (3.0–17.8)
Percentage of BSA change (%), median (IQR)	18.2 (2.2–53.5)	30.3 (6.1–56.3)	25.9 (0.1–54.3)	25.0 (2.3–52.2)	57.1 (14.1–83.6)	63.6 (33.6–85.7)	58.3 (28.3–85.7)	50.0 (25.0–79.1)

MET: metabolic equivalent, representing the rate of energy expenditure while at rest; IQR: Interquartile range; PASI: psoriasis area and severity index; BSA: body surface area.

^‡^ The difference between the patients in different exercise intensity groups in week 8 was statistically significant.

At week 8, the median PASI scores had improved in all groups, ranging from 3.2 (IQR: 1.1–6.5) to 4.1 (IQR: 1.5–7.2). The low-activity group (150 ≤ MET < 500) showed the greatest absolute reduction in PASI score (median reduction: 7.3 points) and the highest median percentage improvement (70.0%). The PASI_50_ response rate at week 8 ranged from 61.2% to 75.1%, with significant heterogeneity among groups (*p* < 0.05). The low-activity group achieved the highest PASI_50_ response rate (75.1%), representing a 13.9-percentage-point absolute increase over the inactive group (61.2%).

BSA scores had also improved by week 8, with median values ranging from 5.0 (IQR: 2.0–11.0) to 7.0 (IQR: 2.6–13.1). The low-activity group exhibited the most substantial absolute improvement in BSA and the highest median percentage improvement, but without statistical significance across the four exercise groups (*p* < 0.05) (Table 2).

### Factors associated with PASI_50_ achievement at Weeks 4 and 8

The univariate LR analysis identified several factors associated with PASI_50_ achievement. Patients in the low-activity group (150 ≤ MET < 500) had significantly higher odds of achieving PASI_50_ at week 8 compared to the inactive reference group (OR: 1.92; 95% CI: 1.13–3.25). Female sex was positively associated with PASI_50_ at both week 4 (OR: 1.34; 95% CI: 1.02–1.75) and week 8 (OR: 1.48; 95% CI: 1.08–2.03). Higher educational attainment (college or above) was significantly associated with increased odds of PASI_50_ at week 4 and week 8. Similarly, patients with monthly incomes of 5001–10,000 RMB (OR: 1.75; 95% CI: 1.17–2.62) and >10,000 RMB (OR: 1.75; 95% CI: 1.07–2.89) had significantly higher odds of PASI_50_ at week 8 compared to the lowest income category (<3000 RMB). Urban residence was associated with improved PASI_50_ outcomes at week 8 (OR: 1.40; 95% CI: 1.07–1.82). Married (OR: 1.98; 95% CI: 1.31–2.99) as well as unmarried (OR: 2.44; 95% CI: 1.46–4.07) patients were more likely to achieve PASI_50_ at week 8 compared to those who were divorced or widowed. In contrast, tobacco smoking was associated with significantly reduced odds of PASI_50_ response at week 4 (OR: 0.82; 95% CI: 0.71–0.94) and week 8 (OR: 0.80; 95% CI: 0.74–0.86). Alcohol consumption was also associated with a lower likelihood of PASI_50_ achievement at week 4 (OR: 0.83; 95% CI: 0.68–1.00) and week 8 (OR: 0.83; 95% CI: 0.74–0.93) (Table 3).

After the adjustment for covariates, patients with low exercise activity (150 ≤ MET < 500) still had significantly higher odds of achieving PASI50 at week 8 (OR: 1.58; 95% CI: 1.01–2.79) compared to the inactive group. The PASI_50_ achievement rate at week 8 among patients with moderate exercise (OR: 1.34; 95% CI: 0.75–2.38) activity as well as high exercise activity (OR: 1.22; 95% CI: 0.61–2.46) were also higher than those in inactive activity group, but without statistical significance (p > 0.05). Interestingly, patients with high exercise activity (MET > 1000) had lower odds of achieving PASI_50_ at week 4 (OR: 0.72; 95% CI: 0.37–1.44) compared to the inactive group, but without statistical significance (*p* > 0.05) (Table 4).

**Table 3.** Influence of different exercise activity intensities on the achievement of PASI_50_ at week 4 and 8 among psoriasis patients characteristics.

Characteristics	Percentage of PASI_50_ at wk 4				Percentage of PASI_50_ at wk 8			
	n (%)		OR (95% CI)		n (%)		OR (95% CI)	
Exercise intensities^‡^								
MET < 150	28	41.8		1.00	41	61.2		1.00
150 ≤ MET < 500	237	44.0	1.09	(0.65–1.83)	405	75.1	**1.92**	**(1.13–3.25)**
500 ≤ MET < 1000	176	43.2	1.06	(0.63–1.79)	284	69.8	1.46	(0.87–2.50)
MET ≥ 1000	37	36.3	0.79	(0.42–1.19)	68	66.7	1.27	(0.67–2.40)
Gender^†‡^								
Male	341	(41.1)		1.00	578	69.6		1.00
Female	138	(48.3)	**1.34**	**(1.02–1.75)**	221	77.3	**1.48**	**(1.08–2.03)**
Age group								
<35 years	134	46.9		1.00	213	74.5		1.00
35–45 years	110	44.0	0.89	(0.63–1.25)	189	75.6	1.06	(0.72–1.57)
46–60 years	108	38.6	0.71	(0.51–0.99)	191	68.2	0.74	(0.51–1.06)
>60 years	127	42.3	0.83	(0.60–1.15)	206	68.7	0.75	(0.52–1.08)
Education^‡^								
Primary and lower	52	36.1		1.00	83	57.6		1.00
Junior high	99	42.7	1.32	(0.86–2.02)	156	67.2	1.51	(0.98–2.32)
Senior high	102	41.0	1.23	(0.80–1.88)	180	72.3	**1.92**	**(1.25–2.95)**
College and above	226	46.0	1.51	(1.03–2.21)	380	77.4	**2.52**	**(1.70–3.72)**
Monthly income (RMB)^‡^								
<3000	54	39.4		1.00	87	63.5		1.00
3000–5000	123	40.1	1.03	(0.68–1.55)	206	67.1	1.17	(0.77–1.79)
5001–10000	235	46.1	1.31	(0.89–1.93)	384	75.3	**1.75**	**(1.17–2.62)**
>10000	67	41.4	1.08	(0.62–1.72)	122	75.3	**1.75**	**(1.07–2.89)**
Residency status^‡^								
Urban	173	42.0	1.06	(0.83–1.36)	277	67.2	**1.40**	**(1.07–1.82)**
Rural	306	43.5		1.00	522	74.1		1.00
Marital status^‡^								
Married	350	42.3	1.05	(0.70–1.58)	599	72.4	**1.98**	**(1.31–2.99)**
Unmarried	85	46.7	1.26	(0.77–2.03)	139	76.4	**2.44**	**(1.46–4.07)**
Divorced/widow/others	44	41.1		1.00	61	57.0		1.00
BMI (kg/m^2^)								
<23.9 (low or normal)	205	45.3		1.00	335	74.0		1.00
24.0–28.0 (overweight)	180	41.2	0.85	(0.65–1.11)	302	69.1	0.79	(0.59–1.06)
>28.0 (obesity)	91	41.9	0.87	(0.63–1.21)	157	72.4	0.92	(0.64–1.33)
Tobacco smoking^†‡^								
Yes	203	38.4	**0.82**	**(0.71–0.94)**	333	63.1	**0.80**	**(0.74–0.86)**
No	276	46.9		1.00	466	79.3		1.00
Alcohol drinking^†‡^								
Yes	73	36.7	**0.83**	**(0.68–1.00)**	122	61.3	**0.83**	**(0.74–0.93)**
No	406	44.3		1.00	677	73.8		1.00
Treatment, (n, %)^†‡^								
Acitretin	12	9.9		1.00	52	43.0		1.00
MTX	87	30.6	**4.03**	**(2.11–7.70)**	179	63.0	**2.25**	**(1.46–3.47)**
NB-UVB	93	39.6	**5.99**	**(3.12–11.49)**	154	65.5	**2.51**	**(1.60–3.93)**
Benvitimod	12	41.4	**6.41**	**(2.48–16.57)**	22	75.9	**4.17**	**(1.67–10.50)**
biologics	271	61.3	**14.23**	**(7.61–26.61)**	387	87.6	**9.39**	**(5.94–14.83)**

MET: metabolic equivalent; OR: odds ratio; CI: confidence interval; BMI: body mass index; RMB: Chinese Yuan.

^†^ The difference in week 4 was statistically significant.

^‡^ The difference in week 8 was statistically significant.The bold values indicate that the difference was statistically significant.

**Table 4.** The influence of exercise activity intensities on the achievement of PASI_50_ at week 4 and week 8 among psoriasis patients based on the multi-variable logistic regression.

Exercise intensities	Achievement of PASI_50_ at wk 4 (OR, 95% CI)			Achievement of PASI_50_ at wk 8 (OR, 95% CI)		
	Model A	Model B	Model C	Model A	Model B	Model C
MET < 150	1.00	1.00	1.00	1.00	1.00	1.00
150 ≤ MET < 500	1.10(0.65–1.86)	1.05(0.63–1.77)	0.88(0.50–1.53)	**1.79(1.03–2.13)**	**1.84(1.07–3.16)**	**1.58(1.01–2.79)**
500 ≤ MET < 1000	1.08(0.63–1.85)	1.02(0.60–1.73)	0.92(0.52–1.62)	1.46(0.83–2.56)	1.42(0.87–2.47)	1.34(0.75–2.38)
MET ≥ 1000	0.82(0.43–1.55)	0.78(0.41–1.47)	0.72(0.37–1.44)	1.28(0.65–2.50)	1.26(0.65–2.44)	1.22(0.61–2.46)

MET: metabolic equivalent; OR: odds ratio; CI: confidence interval.

Model A: Logistic regression with the adjustment of gender, tobacco smoking, alcohol drinking, education, monthly income, residency status, and marital status.

Model B: Logistic regression with the adjustment of gender, tobacco smoking and alcohol drinking.

Model C: Logistic regression with the adjustment of gender, tobacco smoking, alcohol drinking, treatment and GAD-7 score.

### Nonlinear dose-response relationship of exercise activity with PASI_50_ response

**Figure 2.**
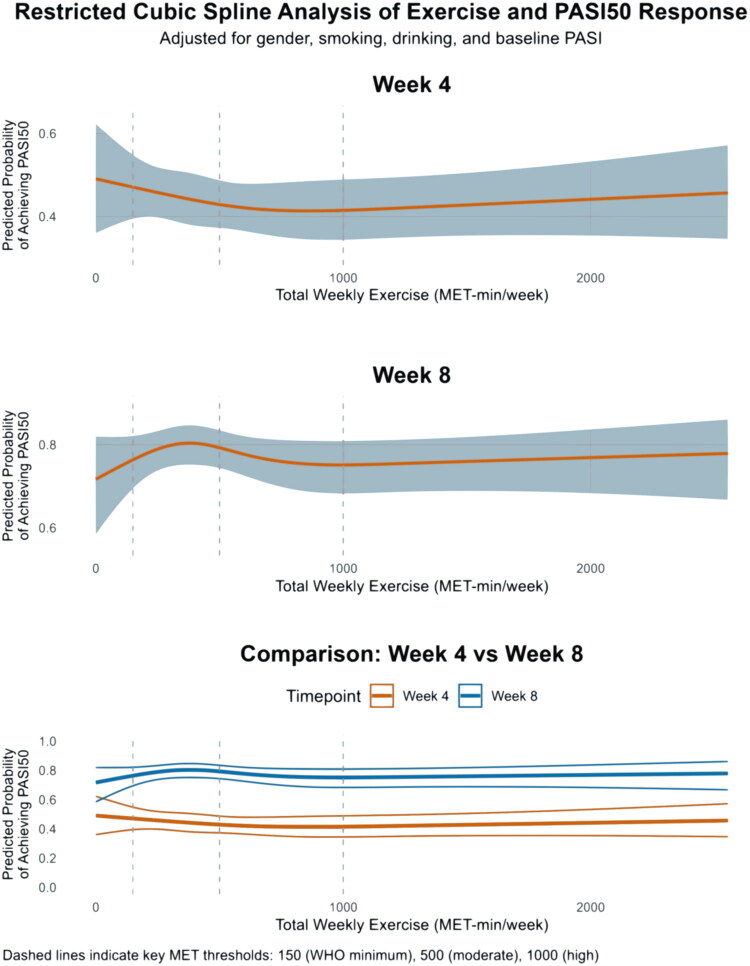
Restricted cubic spline (RCS) analysis of the dose response relationship between weekly (MET-min/week) and PASI50 response at week 4 and week 8 (Adjusted for gender, smoking status, alcohol drinking, baseline PASI score, treatment. Dashed lines indicate key MET thresholds: 150 (WHO minimum), 500 (moderate), and 1000 (high). PASI50:≥50% improvement in Psoriasis Area and Severity Index score from baseline.

The RCS analyses were employed to model the potentially non-linear relationship between the total weekly exercise activity and the predicted probability of achieving PASI_50_ at Weeks 4 and 8, with adjustments for gender, smoking status, alcohol consumption, and baseline PASI score. At Week 4, the association was characterized by a slight initial decline followed by a mild increase across the exercise volume continuum, with wide confidence bands indicating considerable uncertainty. In contrast, the relationship at Week 8 exhibited a clear J-shaped pattern. The probability of achieving PASI_50_ increased rapidly at lower exercise volumes, peaked, and experienced a modest decline, and then gradually stablized. The overall predicted probabilities were higher at Week 8, and the curve at Week 8 demonstrated a pronounced and stable down- ward trajectory beyond the 500 MET-min/week threshold. Moreover, the dashed lines in Figure 2 marking the key MET thresholds (150, 500, 1000) for intuitive reference. The light blue shaded area represents the 95% confidence band, reflecting the uncertainty of the prediction (the narrow confidence band in the 150 ≤ MET < 500 range indicates high reliability of the association, while the wide band in the MET ≥ 1000 range suggests significant uncertainty of the predicted probability in high-intensity exercise group) (Figure 2).

## Discussion

This study observed an association between exercise activity intensity and treatment outcomes in patients with psoriasis: exercise activity within 150 ≤ MET < 500 was linked to more favorable PASI_50_ treatment responses at week 8, while insufficient or excessive exercise was associated with suboptimal responses. Additionally, a potential J-shaped non-linear association was found between continuous exercise activity and PASI_50_ predicted probability, with the highest predicted probability in the low-intensity exercise range (150 ≤ MET < 500). It should be emphasized that this study is an observational study, the results only provide hypothesis-generating evidence for subsequent interventional studies.

Notably, our results challenge the conventional ‘more is better’ approach to exercise prescription. Our analysis indicates that patients engaging in low exercise activity had significantly higher odds of PASI_50_ response compared to those with inactive exercise activity, even after adjusting for key demographic and lifestyle confounders. The higher PASI_50_ response rate in the low-activity group (75.1%) compared with both the inactive (61.2%) and high-activity (66.7%) groups at week 8 suggests that the greatest therapeutic benefit is associated with moderate activity levels, while the advantage diminishes at higher levels. Moreover, the RCS analysis revealed a J-shaped dose-response relationship. From a biological perspective, it is hypothesized that low-intensity exercise may be associated with lower levels of pro-inflammatory cytokines (e.g. TNF-α, IL-6) in psoriasis patients, which may be related to the mild anti-inflammatory effect of moderate physical activity [18,26]. This hypothesis is supported by dermatology-specific research indicating that lifestyle interventions targeting metabolic syndrome can down regulate systemic inflammation, a key driver of psoriatic disease [27]. In contrast, excessive exercise may be linked to increased oxidative stress and hypothalamic-pituitary-adrenal axis activation [26], which may be associated with suboptimal treatment responses. Notably, oxidative stress plays a direct role in the pathogenesis of psoriasis, with studies showing that patients with severe disease exhibit higher serum levels of oxidative stress markers, which could potentially be exacerbated by extreme physical exertion [28]. However, these are only hypothetical mechanistic explanations based on existing literature, and the actual biological mechanisms underlying the observed association need to be explored in subsequent basic and clinical studies.

The observed response pattern also reflects important behavioral and psychological dimensions. Adherence to moderate exercise activity is generally higher than to strenuous regimens, partly due to enhanced self-efficacy and reduced perceived burden [29]. While previous studies have often treated physical activity as a binary exposure, our results underscore the importance of dose-specific recommendations. This nuanced perspective is supported by evidence in autoimmune diseases, where a J-shaped or U-shaped relationship between exercise activity and disease outcomes has been proposed [11]. Our study extends this concept specifically to psoriasis, corroborating recent findings that tailored, non-strenuous physical activity is associated with better disease control and quality of life in psoriatic patients [30].

Beyond exercise activity, our findings encompass broader lifestyle influences. The strong negative association between smoking and PASI_50_ response (adjusted OR: 0.49; 95% CI: 0.36–0.66), along with the adverse trend associated with alcohol consumption, highlights the multifaceted impact of health behaviors on psoriasis outcomes. This finding extends previous observations by Qiang et al.^25^and suggests that smoking cessation may be associated with enhanced association between appropriate exercise and favorable treatment outcomes. The detrimental effects of smoking likely operate through multiple pathways, including direct activation of cutaneous immune cells and promotion of pro-inflammatory cytokines (TNF-α, IL-17, IL-23) that counteract exercise-induced anti-inflammatory effects [31].

Furthermore, the dose-response relationship between educational attainment and treatment success underscores the role of health literacy in disease management. Patients with higher education levels demonstrated significantly greater odds of achieving PASI_50_, likely reflecting better understanding of medical recommendations, enhanced treatment adherence, and improved self-management capabilities [32]. This association emphasizes the need for tailored patient education strategies in psoriasis care.

Several limitations warrant consideration when interpreting the results of this study. First, this is an observational single-center study conducted in Shanghai Skin Diseases Hospital, and the study population is mainly Chinese patients with psoriasis vulgaris in Shanghai, which may limit the generalizability of the results to other groups. Second, the 8-week follow-up period is relatively short, and this study only assessed the short-term treatment response (PASI_50_) of psoriasis. Future studies are needed to verify whether the J-shaped association remains consistent for stricter endpoints such as PASI_75_ and PASI_90_ with longer follow-up and larger sample sizes. Additionally, this study collapsed all physical activities into total MET-min/week, which may obscure differential effects of moderate versus vigorous intensity exercise. Future studies should examine the independent effects of moderate and vigorous physical activity separately to better characterize their respective contributions to psoriasis treatment outcomes. Third, this study did not perform stratification analysis according to different treatment modalities (e.g. topical agents, phototherapy, biologic agents), and the association between exercise activity and treatment outcomes may vary with different treatment methods, which needs to be explored in subsequent stratified studies. Fourth, the physical activity data were obtained through self-reporting by patients, the self-reported nature introduces potential recall and social desirability bias, even with the application of the validated IPAQ-Short Form and face-to-face investigations. Recall bias may occur because patients cannot accurately remember the frequency and duration of their physical activity over the past month, especially for low-intensity and irregular physical activity. Social desirability bias may exist because patients tend to report a higher level of physical activity that is consistent with health recommendations. Fifth, the observational design precludes definitive causal inferences, and residual confounding may persist even after adjusting for multiple confounders.

Despite the above limitations, the study results still have certain generalizability and clinical applicability. First, the study sample size is relatively large (*n* = 1116), and the baseline characteristics of the study population (e.g. age, gender ratio, disease severity) are consistent with those of Chinese psoriasis patients reported in previous epidemiological studies [25], which indicates that the results are relatively representative of Chinese adult psoriasis vulgaris patients. Second, the exercise intensity range (150 ≤ MET < 500) identified corresponds to 20–45 min of daily brisk walking (a low-intensity physical activity), which is easy to implement and adhere to for most psoriasis patients, especially those with mild to moderate disease and no severe joint damage. Third, the study results provide a quantitative exercise recommendation basis for clinicians, who can formulate personalized exercise plans for psoriasis patients according to the MET-min/week index, instead of the traditional vague recommendation of ‘moderate exercise’. Fourth, the finding that excessive exercise is associated with suboptimal treatment responses reminds clinicians to caution psoriasis patients against high-intensity exercise, which has important clinical guiding significance for avoiding ineffective exercise intervention.

Future research should prioritize randomized controlled trials to confirm causal relationships and elucidate underlying mechanisms. Longitudinal studies examining interactions between exercise activity and different treatment modalities, including biologics, would be particularly valuable. Additionally, investigation of molecular mechanisms—including exercise-induced changes in cytokine profiles, oxidative stress markers, and epigenetic modifications—would enhance our understanding of how physical activity influences psoriasis pathophysiology.

## Conclusion

This longitudinal observational study of 1116 patients with psoriasis vulgaris in Shanghai found that exercise activity within the range of 150 ≤ MET < 500 was associated with more favorable short-term treatment outcomes, while insufficient (MET < 150) or excessive (MET ≥ 1000) exercise was linked to suboptimal short-term treatment responses. Our findings suggest that clinicians may consider the necessity of personalized exercise recommendations based on quantitative exercise intensity for psoriasis patients.

## Data Availability

Data for this study are available upon request from the corresponding author. The request should state the title and aim of the research for which data were requested.
